# The strange case of East African annual fishes: aridification correlates with diversification for a savannah aquatic group?

**DOI:** 10.1186/s12862-014-0210-3

**Published:** 2014-10-14

**Authors:** Alexander Dorn, Zuzana Musilová, Matthias Platzer, Kathrin Reichwald, Alessandro Cellerino

**Affiliations:** Fritz Lipmann Institute for Age Research-Leibniz Institute, Jena, Germany; Zoological Institute, University of Basel, Vesalgasse 1, CH-4051 Basel, Switzerland; Scuola Normale Superiore, Pisa, Italy

**Keywords:** Killifish, allopatric speciation, life history evolution, evolution of aging, Africa biogeography

## Abstract

**Background:**

Annual *Nothobranchius* fishes are distributed in East and Southern Africa and inhabit ephemeral pools filled during the monsoon season. *Nothobranchius* show extreme life-history adaptations: embryos survive by entering diapause and they are the vertebrates with the fastest maturation and the shortest lifespan. The distribution of *Nothobranchius* overlaps with the East Africa Rift System. The geological and paleoclimatic history of this region is known in detail: in particular, aridification of East Africa and expansion of grassland habitats started 8 Mya and three humid periods between 3 and 1 Mya are superimposed on the longer-term aridification. These climatic oscillations are thought to have shaped evolution of savannah African mammals. We reconstructed the phylogeny of *Nothobranchius* and dated the different stages of diversification in relation to these paleoclimatic events.

**Results:**

We sequenced one mitochondrial *locus* and five nuclear *loci* in 63 specimens and obtained a robust phylogeny. *Nothobranchius* can be divided in four geographically separated clades whose boundaries largely correspond to the East Africa Rift system. Statistical analysis of dispersal and vicariance identifies a Nilo-Sudan origin with southwards dispersion and confirmed that these four clades are the result of vicariance events In the absence of fossil *Nothobranchius*, molecular clock was calibrated using more distant outgroups (secondary calibration). This method estimates the age of the *Nothobranchius* genus to be 8.3 (6.0 – 10.7) My and the separation of the four clades 4.8 (2.7-7.0) Mya. Diversification within the clades was estimated to have started ~3 Mya and most species pairs were estimated to have an age of 0.5-1 My.

**Conclusions:**

The mechanism of *Nothobranchius* diversification was allopatric and driven by geographic isolation. We propose a scenario where diversification of *Nothobranchius* started in rough coincidence with aridification of East Africa, establishment of grassland habitats and the appearance of the typical African bovid fauna of the savannah. Although confidence intervals for the estimated ages of the four *Nothobranchius* clades are quite large, this scenario is compatible with the biology of extant *Nothobranchius* that are critically dependent on savannah habitats. Therefore, *Nothobranchius* diversification might have been shaped by the same paleoclimatic events that shaped African ungulate evolution.

**Electronic supplementary material:**

The online version of this article (doi:10.1186/s12862-014-0210-3) contains supplementary material, which is available to authorized users.

## Background

Fishes of the genus *Nothobranchius* are annual fishes distributed mostly in East Africa. They are adapted to the alternation of wet and dry seasons and inhabit temporary pools. As a special adaptation to cope with their environment, their eggs have a very hard *chorion*, are resistant to desiccation and hypoxia and undergo diapauses [1–3]. When the habitats desiccate, the adult fish die and the eggs survive encased in the clay during the dry season [4]. Durations of natural habitats can be extremely short: in *N. furzeri*, they were found to last on average 75 days in one season [5]. Under natural conditions, eggs hatch after the seasonal filling of pools by rainfall, though may occasionally be also filled by overflowing rivers [6]. *Nothobranchius* juveniles grow extremely fast and *N. furzeri* can reach sexual maturity in 17 days, the shortest time known for vertebrates [7].

Fishes of the genus *Nothobranchius (N. rachovii*, *N. korthausae, N. guentheri* and especially *N. furzeri)* are emerging as model organisms for genetic and biological studies of aging, as they are the shortest-lived vertebrates that can be cultured in the laboratory and show typical aging markers [8–17]. Different species show remarkable differences in captive longevity: from 3–6 months in *N. furzeri* [18] to 18 months in *N. korthause* [13]. These differences seem to correlate with the habitat conditions, with species from arid habitats (*N. furzeri*) showing faster growth and faster aging than species from humid habitats (*N. korthause* and *N. guentheri*). Differences in aging rates can evolve in relatively fast evolutionary times, as they are observed between closely-related species along an aridity cline [5] and some quantitative *loci* controlling lifespan were mapped [19].

The genus *Nothobranchius* currently includes 62 described *taxa* and several putative species awaiting formal description. These species are distinguished mainly based on the exceptionally diverse and vivid male nuptial colouration; there is relative morphological uniformity among species. The range of the species can be very small and studies in *N. furzeri* suggest a very limited dispersal with prominent isolation by distance and profound geographic structuring [20,21]. Large genetic distances on a small geographical scale were detected also for the species pair *N. kirki*/ *N. wattersi* from Malawi [22]. The range of the genus *Nothobranchius* is limited almost exclusively to the savannah regions of Eastern and Southern Africa and comprises the area between coastal regions in Kwazulu-Natal in the South to Somalia in the North. *Nothobranchius* are also found in the highland plateaus in Zambia, the rift valley, the White Nile region and one population is known from Lake Chad in Central Africa [23]. The hotspot of *Nothobranchius* biodiversity is in the coast of East Africa with 30 of the 62 described species located between Northern Mozambique and Kenya and 10 different species (often with very limited range) within a 200 km radius from Dar-es-Salaam [23]. It is however unclear, whether this hotspot corresponds to the ancestral area of the genus, or whether the species richness of this region is the result of a later dispersal, followed by *in situ* diversification and speciation.

The distribution of *Nothobranchius* largely overlaps with the East African Rift System, a region known to have undergone extensive topographic and climatic remodelling in the Miocene [24] that supposedly caused extensive radiations including the hominoid lineage. There is limited information as to how these events influenced African ichthyofauna and many studies concentrated on the (recent) radiations of cichlid fishes in the rift lakes that is characterized by an early burst of diversification during Plio-Pleistocene (5.3-0.1 Million years ago, depending on the species flock) with subsequent reduced speciation rate due to reduced ecological opportunities [25]. On the other hand, the diversification of the genus *Synodontis*, a *taxon* with panafrican distribution, started in the Miocene (23.3-5.3 Million years ago) in coincidence with major rifting events [26] and shows a constant diversification rate [27]. Annual fishes inhabit savannah pools and may be influenced by the same abiotic factors that shaped diversification of savannah tetrapods. It has been proposed that diversification of African ungulates was strongly influenced by the climatic fluctuations causing cyclic aridification [28]. Since *Nothobranchius* rely on savannah habitats, it is possible that diversification of this *taxon* occurred during arid epoch because of savannah habitats expansion.

Motivation for the present study was to reconstruct a robust phylogeny of the genus *Nothobranchius*, to identify the ancestral area and the patterns of diversification, to investigate possible correlates between separation of different lineages and known geomorphological and paleoclimatic transitions and, specifically, to test whether the diversification rate was constant during the evolution. Finally, we aim to characterize whether adaptation to harsh climate, i.e. short lifespan, is a basal or derived life-history trait in the evolution of the genus.

## Results

### Phylogeny reconstruction

**Figure 1:**
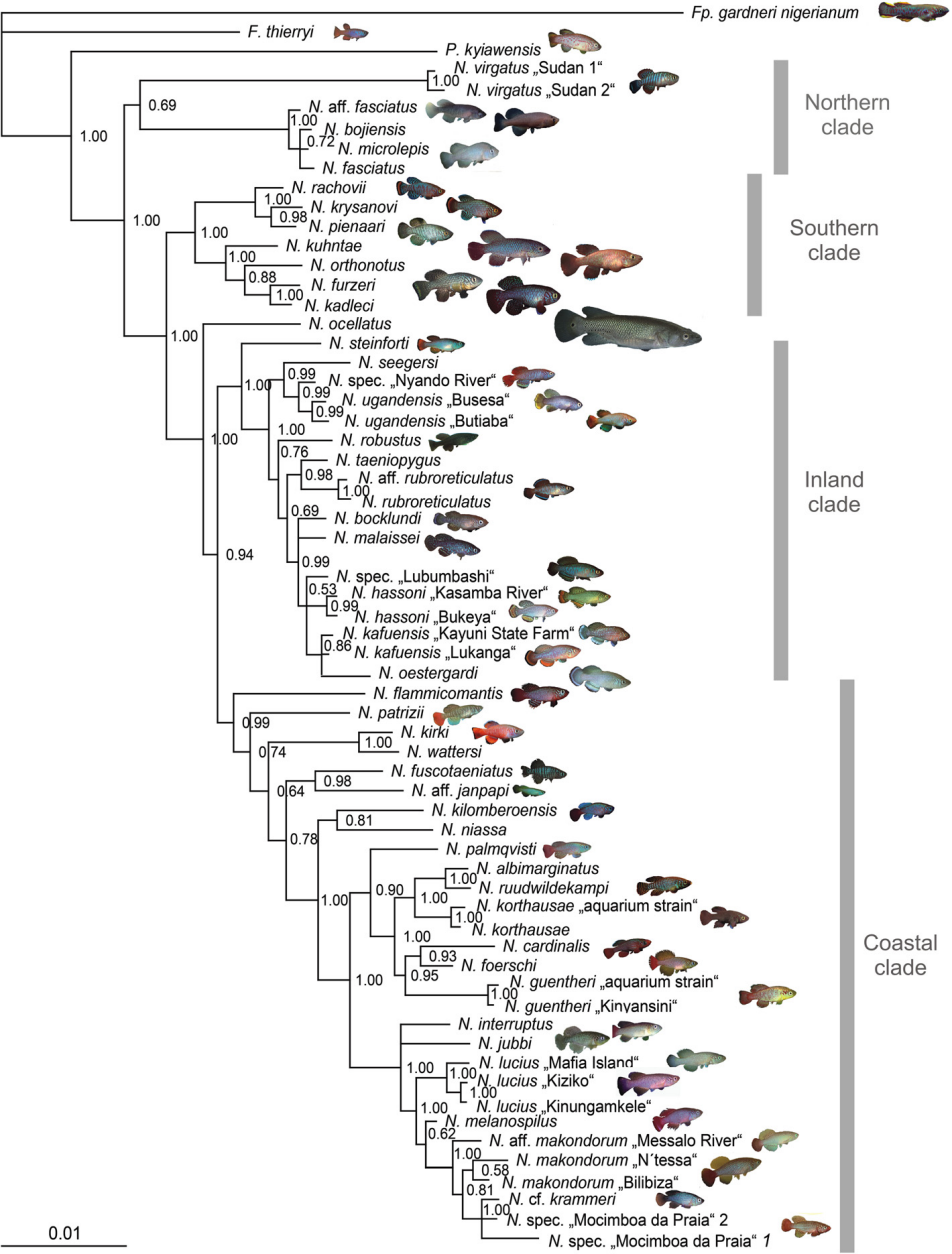
**Phylogenetic hypothesis for** ***Nothobranchius.*** Bayesian phylogenetic tree of the annual killifish genus *Nothobranchius* based on concatenation of partial coding sequences of six genes (*COI*, *GLYT1*, *MYH6*, *SH3PX3*, *GPR85* and *ZIC1*) and fully partitioned dataset. Results of MrBayes run with posterior probabilities shown in the nodes. Four main geographic clades highlighted by the vertical bars.

We analyzed a total of 63 specimens from 46 valid species and four putative species, including three outgroups from West Africa. We used a partial sequence of the mitochondrial *locus* cytochrome oxidase subunit I (*COI)*, and partial sequences of the nuclear genes: Glycin transporter 1 (*GLYT1*), myosin heavy chain 6 (*MYH6)*, SH3 and PX domain containing 3 (*SH3PX3)*, G-protein coupled receptor 85 (*GPR85,* also known as *SREB2*) and zic family member 1 (*ZIC1)* [29] producing a total concatenated sequence of 3847 bp. We found that the substitution rate of the third codon position of the *COI* gene is saturated, and we excluded it from the phylogenetic analyses (Additional file 1: Figure S1). None of the studied *loci* showed individually enough phylogenetic signal (see results of the single-gene analyses in Additional file 2: Figure S2), but the concatenated dataset provided a well-resolved and highly supported phylogeny of the genus *Nothobranchius* (Figure 1). Within the genus *Nothobranchius*, four phylogenetic clades were found by the Bayesian approach. The analysis further revealed that *Pronothobranchius kiyawensis* is the sister *taxon* to *Nothobranchius* (Bayesian posterior probability, BPP = 1) and *Fundulosoma thierry* is a more distant outgroup. *Nothobranchius* is clearly monophyletic with BPP = 1 and the basalmost *Nothobranchius* clade is a Northern clade that consists of the species *N. virgatus* from White Nile region and the *N. microlepis* species complex from Somalia/Northern Kenya, although with moderate support (BPP = 0.69).

All other species of *Nothobranchius* are grouped in three highly-supported clades that are strictly separated by geographic barriers (Figure 1): 1) the Southern clade contains all species found south of the Zambezi and is sister to the rest of *Nothobranchius*. Two species-rich sister clades – i.e. 2) the Inland- and 3) the Coastal clades contain all species from the inland plateaus, and all species from the coastal habitats North of Zambezi, respectively (Figure 1). The Southern clade (BPP = 1) comprises the *N. orthonotus* species complex and the *N. rachovii* species complex.

The Inland- and Coastal-clades represent sister groups together with the large predatory species *N. ocellatus* (BPP = 1) that appears as an isolated basal form to these two clades. The Coastal clade (BPP = 0.99) shows a clear structure with two basal isolated species: *N. flammicomantis* and *N. patrizii,* two species pairs: *N. wattersi*/*N. kirki* (BPP = 1) from the Lake Malawi system [22] and *N. fuscotaeniatus/N. janpapi* from coastal Tanzania (BP = 0.96). Further, a strongly supported clade (BPP = 1) contains 15 species and some putative species from the Southern edge of distribution of the Coastal clade. A separate lineage within this clade is formed by the *N. kilomberoensis/N. niassa* species pair (BPP = 0.81) from Northern Mozambique and Southern Tanzania [30]. Then, two major, strongly-supported groups are recognized: one includes larger and more elongated species (e.g. *N. melanospilus* species complex and *N. jubbi* and *N. interruptus* from Somalia and Kenya; [31]) and the other group (BPP = 1) includes small species from coastal Tanzania and the islands of Mafia and Zanzibar. The distribution of these two groups overlaps to a large extent. The Inland clade (BPP = 1) is geographically-structured and comprises the well-differentiated *N. seegersi* species complex (BPP = 0.99) that spans from Northern Lake Malawi to Southern Lake Victoria, and the *N. taeniopygius* species complex, that includes the very broadly distributed species *N. rubroreticulatus* and a group of species from Kafue basin and neighboring regions (*N. kafuensis* species complex)*.*

The four *Nothobranchius* phylogenetic clades show strict geographic segregation. The most basal taxa live in the Northern edge of the genus range (Northern clade), the Southern clade is geographically isolated from the rest of species and the two species-rich Coastal and Inland clades are distributed in the middle of the range. There is almost no overlap in the range of these clades, except for some limited examples in the Northern edges of the range. In the Kenya coastal drainages, there is an overlap between the Northern (basal) clade and the Coastal clade: a population of *N. bojiensis* occurs in sympatry (syntopy) with one population of *N. jubbi*. In the White Nile region, there is an overlap between the Northern (basal) clade and the Inland Clade: a population of *N. virgatus* occurs close to *N. rubroreticulatus*.

### Biogeographical analysis

**Figure 2:**
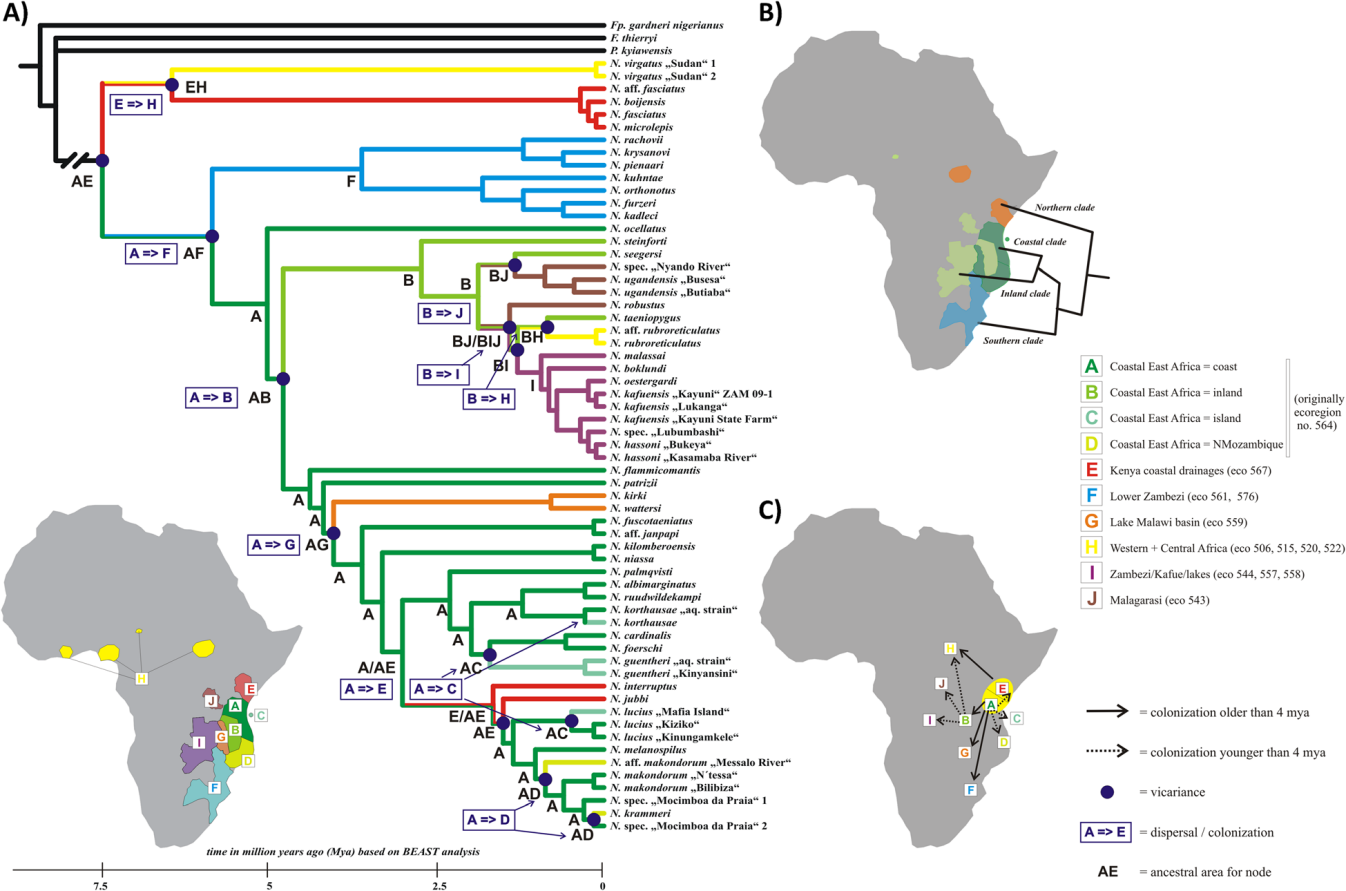
**Results of biogeographic analysis.** Historical biogeography reconstruction using the Statistical Dispersal-Vicariance Analysis (S-DiVA) **A)** Topology of the bayesian phylogenetic tree with mapped distribution area and dispersal & vicariance events. Age of nodes with vicariant event corresponds to the timeline (after BEAST analysis). A-J represents the biogeographic regions used for S-DiVA analysis based following with slight modifications) the proposed African freshwater ecoregion (Abell et al., [32]). See the unit description in the legend on the right side of the figure, as well as on the schematic Africa map. **B)** The schematic graphics of the four *Nothobranchius* clades and their allopatric distribution. **C)** The proposed colonization events older than 4 Mya (bold line) and younger than 4 Mya (dashed line).

Statistical Dispersal-Vicariance Analysis (S-DIVA) on basis of 10 ecoregions (Figure 2) demonstrated that East African coast (region A – Coastal East Africa – coast) and Kenya (region E – Kenya coastal drainages) represent the ancestral area for all the extant *Nothobranchius* species, from where the ancestors colonized the rest of the recent distribution area (Figure 2).

Thirteen dispersal (=colonization) events mostly followed by the vicariance were detected within the evolutionary history of *Nothobranchius* and provide explanation of the contemporary distribution pattern (Figure 2).

The first vicariance event occurred in the ancestral area (region A + E) between the Northern (region E) and Eastern coastal (region A) ancestors giving rise to the separate basal Northern clade and followed by the colonization (=dispersal) from the coast (region A) to the South (region F). After that, vicariance caused the separation of the Southern clade from the coastal forms. Later, another colonization took place from the coast (region A) towards the inland part of the continent, reaching the Inland Plateau first (region B) and following to the other inland habitats from there, suggesting a scenario with a single colonization wave to the Kafue and Zambezi river systems (region I), two waves to the Malagarasi system (region J), and one colonization even far Northeast to the White Nile region and lake Chad (region H; *N. rubroreticulatus*). (See Figure 2A, C). Another independent colonization from the coastal region (region A) to the rivers of Lake Malawi basin (region G) and back to the Northern coastal region into Kenya (region E) gave rise to secondary contact with the basal *Nothobranchius* clade and cases of recent sympatry. Finally, the most recent colonizations happened from the coastal region (region A) to the islands of Zanzibar (*N. guentheri, N. melanopilus*) and Mafia (*N. korthausae, N. lucius;* both islands region C), and of the *N. melanospilus* lineage to the south along the coast into Northern Mozambique (region D).

### Molecular clock dating and test for shifts in diversification rate

The dating of diversification events was estimated using relaxed molecular clock analysis. In the absence of suitable fossils, we adopted a secondary calibration strategy based on the most recent dated teleost phylogeny [33] that includes several members of Cyprinodontiformes, but not *Nothobranchius*. We first performed a molecular clock analysis that includes members of Cyprinodontiformes for which estimated ages are available and a small subset of *Nothobranchius* species representative of the four different clades (Additional file 3: Figure S3) and estimated the age of *Nothobranchius* genus to be 8.32 (CI 5.92 – 10.75) My. We then used this value as a secondary calibration for *Nothobranchius*-focused analysis. Based on the results, the separation of the Southern clade was dated 5.99 (CI 3.38-8.48) Mya and the separation of Coastal and Inland clades was dated 4.85 (CI 2.76-7.00) Mya. Between 3 and 4 Mya, diversification occurred in all three clades with 1) separation of the *N. rachovii* and *N. orthonotus* clades in the Southern clade, 2) divergence of all basal isolated clades in the Coastal clade followed by divergences of the *N. melanospilus* and *N. guentheri* species complex, and 3) separation of the isolated *N. steinforti* from all other taxa of the Inland clade.

**Figure 3:**
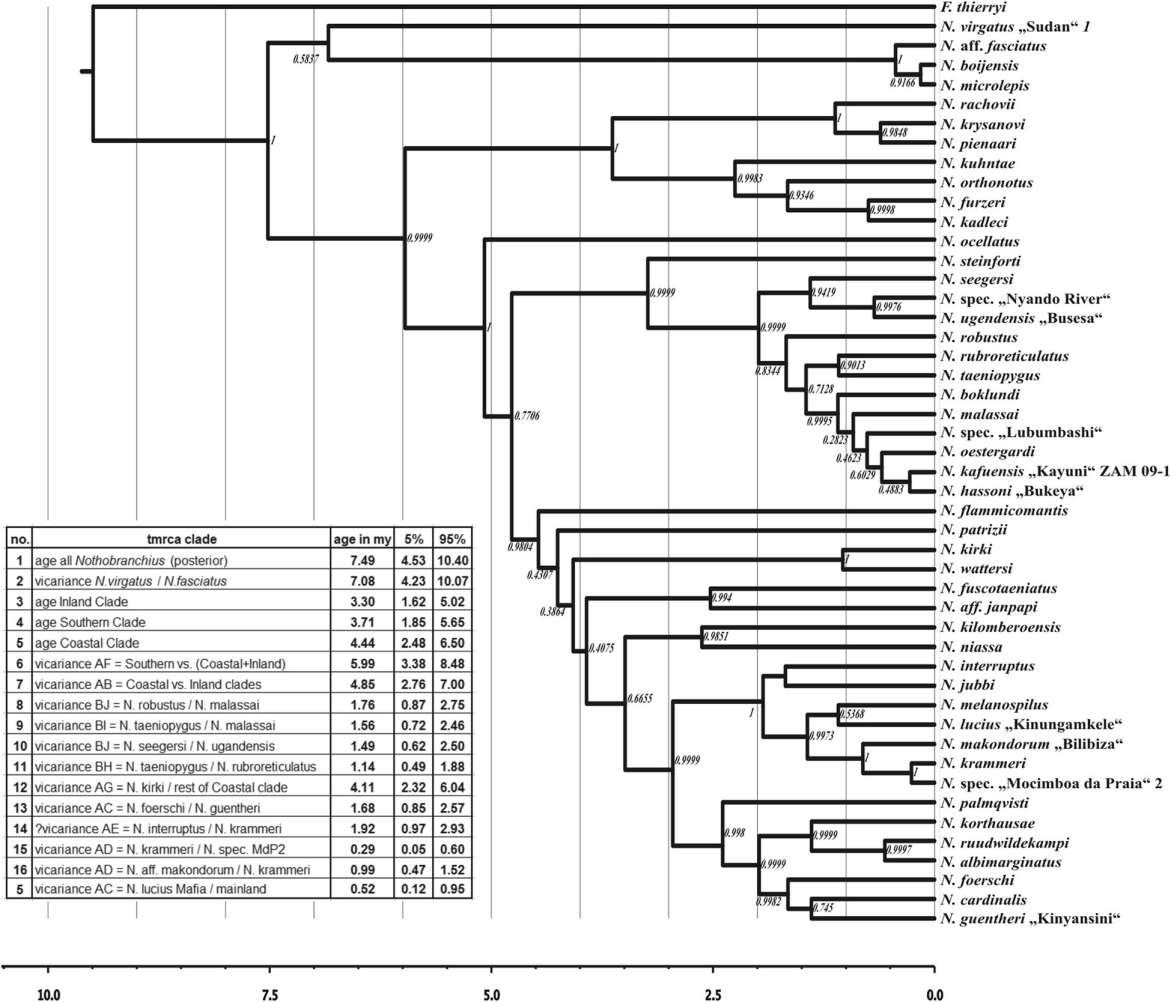
**Calibrated phylogenetic tree.** Molecular clock tree reconstructed by BEAST analysis. The tree was calibrated by the secondary calibration of 8.32 (5.92 – 10.75) Mya for the genus *Nothobranchius*. Time axis shows the age in mya. The table embedded within the image reports the age estimates of individual nodes in the molecular-clock tree, where vicariance or dispersal occured. Results from the analysis in BEAST software using one individual per species.

The final step of diversification was the divergence of several pairs of closely related sister species (*N. rachovii* and *N. pienaari*, *N. furzeri* and *N. kadleci*, *N. oestergaardi* and *N. kafuensis*, *N. ugandensis* and *N. sp.* “Lake Victoria”, *N. hassoni* and *N. sp.* “Lubumbashi”, *N. kirki* and *N. wattersi*, *N. melanospilus* and *N. lucius*, *N. ruudwildekampi* and *N. albimarginatus*) that occurred between ~ 0.5 and ~ 1 Mya. (Figure 3). We specifically tested for deviation from constant rate of cladogenesis by analysing lineage through time using TreePar [34] but could not detect deviations from a constant cladogenesis rate (Additional file 4: Table S3).

## Discussion

In this study, we report the first comprehensive phylogenetic investigation of the genus *Nothobranchius*. We used extensive taxon sampling and combination of mitochondrial- and nuclear markers along with statistical analysis of dispersal and vicariance events and diversification rate to reconstruct the biogeographic history of *Nothobranchius*. In the absence of any paleontological calibration points, we also provided molecular-clock dating based on secondary calibration.

The main results are a robust phylogeny of the genus *Nothobranchius* and a tentative dating for diversification events. Four robust geographic clades were recovered: a basal Northern clade, a Southern clade (distributed south of the Zambezi River), an Inland clade (distributed in the East African plateau) and a Costal clade (corresponding to East African coast). As all the four clades have almost exclusively allopatric distribution (with only small overlap by Coastal and Northern clade in coastal Kenya, due to secondary contact) and, because there are no examples of sister species with overlapping ranges and many examples of sister species with neighboring ranges, we conclude that the main mechanism of diversification in *Nothobranchius* was allopatric speciation driven by geographic isolation.

Some results were unexpected and joined in pairs species with little morphological similarities: e. g. *N. virgatus* and the *N. microlepis* species complex or *N. fuscotaeniatus* with *N. janpapi*. In general, however, the relationships are consistent with previous morphological studies [23]. In case of *N. virgatus + N. microlepis*, their sister position could be explained by the artefact of the long-branch attraction although we followed the instruction to select correct model of evolution. The signal comes mostly from the *COI* gene (Additional file 2: Figure S2), in the more conserved nuclear genes, such sister position has not been observed. However, if not sister, these lineages belong to the base of the tree.

We tried to provide a timing for these biogeographic events. Unfortunately, there is no fossil of *Nothobranchius* available, so the fossil calibration was not the option for the present study. Further, we considered the predicted substitution rate of the *COI* gene. The rate could have been applied from the lifebearers (family Goodeidae), phylogenetically not very distant group from *Nothobranchius* (both belonging to the order Cyprinodontiformes), where the rate has been calculated as 0.0045 substitution per site per My (s/s/My: [35]) However, annual fishes have evolved a peculiar mitochondrial physiology in order to cope with anoxia during diapause when mitochondria are not poised to produce ATP, but rather to shuttle carbon and electrons through the Kreb’s cycle while minimizing the generation of a proton motive force [36]. The evolutionary constraints on the protein-coding part of the mitochondrial genome (including the *COI*) differ between annual- and non-annual Cyprinodontiformes. Furthermore, even within lifebearers, in the genus *Poecilia*, the mutation rate was found to be three-fold slower (0.00145; [37]). Finally, COI shows saturation in our dataset and is therefore suboptimal as molecular clock. Thus, there is no reliable substitution rate applicable to the genus *Nothobranchius*. Therefore, we had to use a secondary calibration strategy for the molecular clock using as calibration more distant outgroup *taxa* within Cyprinodontiformes [33].

The results of this analysis have to be dealt with caution, but are, at present, the only option available. The separation of the four main clades is the result of vicariance events. Using secondary calibration, it is estimated that the age of the *Nothobranchius* genus corresponds to the late Miocene, but diversification within the Inland and Costal clades resulted from several later dispersal and vicariance events in the Pliocene and early Pleistocene. The extant species of *Nothobranchius* are estimated to have an age of 0.5-1 My and therefore climatic oscillations during the late Pliocene likely resulted in intraspecific diversification rather than speciation [20]. *Nothobranchius* fishes seem to represent older assemblage than other fish *taxa* such as lacustrine cichlids or sticklebacks that are currently model organisms to investigate the genetic architecture of diversification [25,38–42] and their timing of diversification seems to be more in pace with that of the slower-evolving African teleost genus *Synodontis* [27].

### Phylogeographic view of *Nothobranchius:* the role of tectonics and paleoclimate

Our analysis clearly supports the monophyly of *Nothobranchius*. The sister *taxa* of *Nothobranchius* are found in West Africa, where also many non-annual (genus *Aphyosemion*) and facultative-annual (genus *Fundulopanchax*) species of Cyprinodontiformes are found [43]. It is trivial that the most recent ancestor of *Nothobranchius* originated in West Africa, but it is surprising the absence of *Nothobranchius* from Central Africa. Probably, the alternation of African climate resulted in periods when annual fish habitats were restricted causing thereby potential extinction of the basalmost forms in Central Africa and the presence of the equatorial belt of pluvial forest restricts current *Nothobranchius* distribution. *N. rubroreticulatus*, the only *Nothobranchius* species found in Central Africa [23], is a member of the Inland Clade and showed a secondary dispersion with a peculiar disjunct distribution with one population in Lake Chad and one in the White Nile.

We tried to put the reconstruction of *Nothobranchius* phylography in the frame of evolution of African topography, climate and vegetation in the Neogene. Five events in the evolution of East Africa are of particular relevance to the *Nothobranchius* diversification: i) the formation of the East Africa Rift System and the uplifting of Ethiopian plateau ~20 Mya followed by ii) the uplift of the East African plateau ~13 Mya iii) the so-called Miocene climate optimum (11–17 Mya) characterized by high precipitations [44] followed by iv) aridification of East Africa starting ~ 8 Mya and ripe by 5 Mya with progressive restriction of forest and expansion of savannah habitat [45,46] that was followed by v) alternation of more arid and more humid epochs [24].

We propose a *scenario* of *Nothobranchius* diversification that results from the combination of molecular clock- and biogeographic analysis. The ancestor of *Nothobranchius* dispersed from West Africa through Central Africa and the Nilo-Sudan region and then Southwards and inwards. The age of *Nothobranchius* was estimated in 8.32 (5.92 – 10.75) My. This epoch is clearly later than the formation of the East African Rift and yet the first phase of *Nothobranchius* diversification is characterized by vicariances along lines that roughly correspond to the East African Rift System. We cannot offer an explanation for this discrepancy, but wish to remark that also in cichlid fishes there is a discrepancy between geological events and molecular clock analysis: the estimated age of the most common ancestor of the African and Southamerican cichlid clades is much younger than Gondwana separation [47]. The first vicariance separated the two species of the basal Northern clade from the common ancestor of all other lineages in the Rovuma Plate with Southwards dispersion and further separation of the *N. microlepis* species group in the Somalian plate from *N. virgatus* in the Nubian Plate. The second vicariance event separated the Southern clade and we dated this divergence ~ 6 Mya (CI 3.4-8.5). It should be noted that there is also an apparent lack of extant *Nothobranchius* between Northern Mozambique and the Malawi Rift, which corresponds to the predicted colonization route by ancestors of the Southern Clade. This area is currently covered by forest [48] and represents a barrier for annual savannah fishes. It is interesting to mention that this same region represents a suture zone for some savannah mammals [28] (see next paragraph).

A third inwards dispersion resulted in inland colonization followed by a third vicariance event that separated the species from the East African plateau resulting in the separation of the Inland and Coastal clades along the Tanzanian section of the East African Rift. We dated this separation as 4.8 My (CI 2.7-7.0). The relatively large confidence intervals of these events make it difficult to pinpoint specific climatic changes, but two general conclusions can be drawn: i) origin of *Nothobranchius* and early diversification overlap with aridification of East Africa and appearance of grassland habitats [49], ii) the vicariance lines of the different clades correspond largely to the sections of the East African Rift system but appear to be more recent than initial rifting. It is well established that an alternation of wet and dry periods characterized at least the last 3 million years of East Africa as evidenced by the repeated filling and drying of the Rift Valley Lakes [24]. In particular, three humid periods at 2.7 to 2.5 million years ago (Mya), 1.9 to 1.7 Mya, and 1.1 to 0.9 Mya, superimposed on the longerterm aridification of East Africa, were detected [24]. It is logical to speculate that these climatic oscillations shaped the timing of *Nothobranchius* diversification with dispersals (and diversification) occurring during arid periods and stasis during humid periods. It should be noticed that the end of the last humid period described by Trauth et al. [24] ~ 1 Mya corresponds to the estimated diversification of many pairs of *Nothobranchius* sisters species (*N. oestergaardi* and *N. kafuensis*, *N. ugandensis* and *N. sp.* “Lake Victoria”, *N. hassoni* and *N. sp.* “Lubumbashi”, *N. ruudwildekampi* and *N. albimarginatus* and differentiation within the *N. melanospilus* clade). This period also corresponds to the middle Pleistocene transition with the emergence of low-frequency, high-amplitude, quasi-periodic (∼100-kyr) glacial variability [50].

With all the cautions needed given the large confidence intervals, it seems therefore that the alternation of glacial and interglacial periods during the late Pleistocene did not lead to speciation, but rather to geographic substructuring of extant species due to isolation by distance with little morphological diversification, as exemplified by the *N. furzeri* species complex [20].

### Comparison with other *taxa*

A key issue in phylogeography and biogeography is the concordance of geographic patterns across *taxa. Taxa* showing concordant phylogeographic patterns are likely to be shaped by the same biotic or abiotic factors. In this respect, two studies are relevant to *Nothobranchius*: i) continent-wide investigations of freshwater fishes that were likely shaped by tectonics, and ii) studies of savannah vertebrates that were likely shaped by aridification and expansion of grassland. One of the few available studies on African riverine fish with extensive taxonomic and geographic sampling is focused on the genus *Synodontis* [27]. The genus *Synodontis* is inferred to have originated in the broad region of West Africa and the clade that gave rise to East African colonization is specifically associated with Nilo-Sudan region with Southward dispersion. Dating in this study is based on the fossil record and diversification of *Synodontis* seems to be more ancient than *Nothobranchius* early vicariances. The timing of the divergence between the Nilo-Sudan and East-African *Synodontis* clades is compatible with the upsurge of the Ethiopian plateau (95% HPD 18.1-26.7 Ma). Diversification of the East African biogeographic clade (95% HPD: 8.6-14.3 Ma) is compatible with uplift of the Central Plateau.

On a larger phylogenetic distance, the African ungulates (especially bovids) represent a *taxon* with comprehensive biogeographic information available. The emergence of this group is temporally linked to the emergence of grassland habitats during the Pliocene and appearance of many bovid *taxa* in the fossil record is dated around 3 Mya [51,52]. This timing is consistent with the median estimated age of the three main *Nothobranchius* clades (Southern clade: 3.7 My CI = 1.8-5.6, Inland clade: 3.30 My CI = 1.6-5.0, Costal clade: 4.4 My CI = 2.4- 6.5). Ungulates and killifishes share the dependence on savannah habitats and it is natural to speculate that their initial diversification occurred in the same epochs. However, it is believed that the alternation of pluvial cycles was the driving force in diversification of large herbivores in late Pleistocene. As detailed above, *Nothobranchius* species seems to be already differentiated during this era and climatic alternations likely resulted in their strong geographic substructing [20].

There is concordance between *Nothobranchius* and ungulate biogeographic patterns. Several species show subcontinent-wide distribution and clear genetic structuring. Further evidence shows that the separation between a Nilo-Sudan and East-South African lineages is a robust signal observed not only in *Nothobranchius* or ungulates, but also across other studied *taxa* such as ostrich [53] or white-tailed mongoose [54]. This genetic structuring is likely due to the equatorial forest belt that represents a barrier to dispersal. Further, for some species (Common eland, Common warthog, Hartebeest, Sable; reviewed in [28]) a *suture zone* is observed between an East African and a South African genetic group(s) that broadly corresponds to the border between the Southern- and East African clades in *Nothobranchius*. The existence of such *suture zone* is indicative for existence of separate *refugia* during the pluvial periods followed by secondary contacts after savannah expansion during arid periods in Pleistocene.

Therefore, the extensive studies on African ungulates reveal that observed the borders of the *Nothobranchius* clades (this study), which were likely established between the late Miocene and early Pleistocene, persisted (or reappeared) as barriers that have hindered dispersal of savannah mammals up to the present.

In summary, in light of the broad biogeographic pattern of *Nothobranchius*, molecular clock analysis and the dependence of extant *Nothobranchius* on savannah habitats, we propose a scenario where aridification promoted diversification of this aquatic *taxon*.

### Basal and derived forms within the genus *Nothobranchius*

The comprehensive phylogenetic hypothesis produced within this study allows us also to interpret the ancestral and derived states either in morphological or in the life-history traits. Fishes from the genus *Nothobranchius* show relative morphological uniformity, with the exception of three lineages that can be immediately separated by their morphology. First, the *N. microlepis* species complex is characterized by very small scales, drab color, small eyes and a different mating behaviour. Second, *N. ocellatus* is characterized by a very large size, elongated shape and the typical *habitus* of a lurking predator. And third, the subgenus *Aphyobranchius*, of which we analyzed the species *N. janpapi,* is characterized by small size and the typical shape of a surface-oriented fish [23]. Our analysis groups *N. microlepis* species complex with *N. virgatus* although with moderate support and we are unable to identify the ancestral morphotype. For the other two lineages, we can conclude that they represent derived forms that likely evolved to enter novel ecological/trophic niches. Most of the *Nothobranchius* species typically feed on invertebrates that are present in the water column [55], while *N. ocellatus* is the only ichthyophagous *Nothobranchius* and the subgenus *Aphyobranchius* have the typical morphology of surface-prey oriented fish. Suprisingly, *Aphyobranchius* are a sister taxon to *N. fuscotaeniatus*, with which they share only little morphological similarity.

A substantial variation in the climatic conditions is observed across *Nothobranchius* habitats. Although all *Nothobranchius* are annual fish, some species are found in semi-arid habitats with as little as 400 mm/year and a prolonged dry season, while others are found in habitats with two rainy seasons with longer-lasting habitats (Watters [31]). The duration of the habitats sets a limit to maximum survival and drives evolution of life-history traits: fish from arid habitats (such as *N. furzeri*) show longer diapause, rapid growth and short lifespan, while fish from humid habitats (such as *N. korthause*) show shorter diapauses, slower growth and longer lifespan [13]. Climatic differences were shown to induce parallel evolution of aging rate in pairs of closely related species of the Southern clade [5]. Our results show that all species of the basal Northern clade are found in arid environments such as Somalia and the White Nile region in Sudan and are therefore short-lived in their natural habitat (Figure 1). The Southern clade also contains species found in the arid bushveld of Southern Mozambique that are short-lived both in the wild and in captivity [5]. All species from the coastal clade, on the other hand, originate from humid habitats and are expected to be longer-lived, as confirmed in captivity for *N. korthause* [13] and *N. guentheri* [12]. This suggests that the basal form of *Nothobranchius* was likely adapted to an arid environment, while the longer-lived forms are representatives of a derived life-history trait. This is consistent with a more general picture of the evolution of diapause and annualism in Cyprinodontiformes [56] that demonstrated how annualism is an ancestral trait that was lost three times and regained once [56]. This suggests that such life-history adaptations can rapidly undergo evolutionary changes when the selective pressure of arid environment is relaxed.

## Conclusions

We conclude that the *Nothobranchius* genus seems to be an old assemblage and origin of the genus was estimated during the late Miocene. Diversification of *Nothobranchius* was characterized by West-African origin, a transition through the Nilo-Sudan area and a Southwards dispersion. The main four *Nothobranchius* clades were established by vicariance events along lines that roughly correspond to the East African Rift system. Diversification was estimated in rough coincidence with the progression of East Africa aridification. Therefore, *Nothobranchius* phylogenetic history seems to have been influenced by geological events that shaped evolution also of other fish assemblages, but also by paleoclimatic transitions (aridification) that shaped evolution of savannah tetrapods.

## Methods

We performed phylogenetic analysis based on an extensive taxonomic sampling of the genus *Nothobranchius*, and on mitochondrial (*COI*) and five nuclear loci (*GLYT1*, *MYH6*, *SH3PX3*, *GPR85* and *ZIC1*) originally selected by [29] and are now widely used for fish phylogenetics [33].

### Taxon sampling and DNA-extraction

The genus *Nothobranchius* is distributed over a large part of East and Southern Africa, sometimes in regions that are of difficult access for geopolitical reasons (e.g. Somalia). Since extensive sampling of the entire distribution range was not a feasible option, we took advantage of a large community of specialized and highly-dedicated breeders that have as mission the maintenance of pure genetic strains, the vast majority of these captive strains are endowed with a collection code that identifies the original collection locality [4]. These dedicated hobbyists are also responsible for almost all formal descriptions of Nothobranchius species. The samples used in this study, all originate from captive specimens that were either part of one of the author’s (AD) private collection or were provided by specialized breeders who are expert in *Nothobranchius* taxonomy (see Acknowledgments). In all cases, the tissue was preserved in 98% ethanol. A summary of the specimens with collection codes, the locality and (where available) the GPS coordinates is provided in Additional file 5: Table S1. The samples are deposited in the “Museo di Storia Naturale e del Territorio” of the University of Pisa and the sample numbers are indicated in Additional file 5: Table S1. For DNA-extraction fin clips were used as previously described [21].

### Primers used in the study

Several *Nothobranchius*-specific primers were designed within this study. First, for the *COI* gene, first set of primers was designed by aligning the complete sequence of *COI* from *N. furzeri* (NC_011814.1) with that of two other Cyprinodontiformes, *Kryptolebias marmoratus* (NC_003290.1) and *Cyprinodon rubrofluvitalis* (EF442803.2), in order to identify conserved regions. As these primers often failed to amplify the *COI* gene of *Nothobranchius*, a second set of primers was designed by aligning the *COI* sequences of *N. furzeri*, *N. rachovii*, *N. pienaari* and *F. thierryi* obtained from the previous set of primers in order to obtain *Nothobranchius*-specific primers.

The primers used for amplifying the nuclear marker genes with nested strategy were described [29]. Sometimes these primer sets failed to amplify the genes, too. Alignments of the sequences obtained from *N. furzeri*, *N. rachovii*, *N. korthausae* and *N. kuhntae* were used for designing a second set of *Nothobranchius*-specific primers for the second PCR-reaction. The list of primers, as well as the position of *COI* primers along the gene is shown in Additional file 6: Table S2.

### PCR-reactions and sequencing

PCRs were performed at 25 μl final volume, each with 2.5 μl 10x PCR buffer; 1.5 μl 25 mM MgCl2; 0.5 μl each of 10 mM dNTP mix, 10 μM forward, 10 μM reverse primers, 0.25 μl 5 U/μl Taq Polymerase (Qiagen) and 100-150 ng of genomic DNA using an EPPENDORF thermocycler (Mastercycler ep gradient s). The PCR-program for primers 22f-23r and 20f-3r was: 94°C for 120 s followed by 10 cycles with a touchdown (94°C for 30 s, touchdown from 55°C to 50°C, 0.5°C decrease at every step) and 30 cycles (94°C for 30 s, 50°C for 30 s, 72°C for 90 s), and a final 180 s at 72°C. For primers 19f-21r, the same program was used except that touchdown was performed from 51°C to 46°C and the annealing temperature was 46°C. For primers 132f and 136r, the following program was used: 94°C for 120 s, 35 cycles (94°C for 30 s, 57°C for 30 s and 72°C for 30 s) and 72°C for 60 s. Sequencing was performed using the BigDye Terminator v3.1 Cycle Sequencing Kit (ABI; Weiterstadt, Germany), followed by separation on ABI 3730xl capillary sequencers. After quality clipping, sequences were assembled based on overlaps using the GAP4 module of the Staden Sequence Analysis Package as described previously [57].

### Phylogenetic and molecular clock analysis

Sequences of the studied markers were then aligned using ClustalW as implemented in the BioEdit software package http://www.mbio.ncsu.edu/bioedit/bioedit.html). Data sets for particular genes were analyzed separately, as well as we performed the overall analysis of the concatenated data set including all the studied markers. All the single-gene alignments as well as the concatenated alignment were exported to the nexus format for following phylogenetic analyses. Mitochondrial gene (*COI*) was tested on saturation by plotting the genetic distance under the selected model (GTR + I + gamma) against the uncorrected p-distance for each codon positions. The deviation from the linear ratio in the plot for the third codon position was visually evaluated.

Bayesian approach was selected to reconstruct phylogeny using MrBayes 3.1 software [58]. Parameters for the analysis were set based on the suggested models of evolution by jModeltest [59]. The data set was partitioned by *loci*, and additionally the COI gene was partitioned by codon position. The mcmc analyses were then set to run for 5 million generations for the single gene data sets, and 10 million generations in case of the concatenated data set, respectively. The parameters were unlinked for each gene partition within the concatenated data set to be considered independently.

We further performed the relaxed Molecular clock analysis in BEAST software [60] under the uncorrelated log-normal clock settings, birth-death model and 10 million generations in two independent runs. Data set was partitioned by *loci*, and by codon position in COI. Only one individual per species has been used to build such tree to avoid artefactual signal coming from intraspecific relationships. Because no fossil record is available for *Nothobranchius*, we used the secondary calibration approach. First we performed an additional molecular-clock analysis based on a restricted subset of *Nothobranchius* (10 species across the phylogeny) and including seven representatives from families Cyprinodontidae, Poecilidae, Fundulidae and Aplocheilidae, for which sequences of the same markers used herein are available [33]. Only the nuclear genes were used to build this supportive tree (the COI marker in Near et al., [33] covered different part of a gene than this study) and we calibrated this “Cyprinodontiformes” tree by the known node ages from the large-scale fish phylogeny calibrated by 37 fossils [33].

We then estimated the age of the *Nothobranchius* genus and used it as a secondary calibration for the *Nothobranchius* tree. Five calibration points were used for the “Cyprinodontiformes” analysis: *Poeciliopsis* + *Gambusia* split in 13.5 mya, *Cyprinodon* + *Jordanella* split in 21 Mya, *Fundulus* + *Lucania* split in 17.5 mya, (*Poeciliopsis*, *Gambusia*) + (*Fundulus*, *Lucania*) split in 44.5 Mya and (*Cyprinodon*, *Jordanella*) + (*Poeciliopsis*, *Gambusia*, *Fundulus*, *Lucania*) split in 51 Mya. The calibration point for the *Nothobranchius* molecular clock was set to 8.32 mya for the first split within the genus. For both molecular-clock analyses we used the normal distribution to set the calibration prior, with the mean at the estimated age, and the 95% interval included following the range provided by Near et al. [33].

### Biogeographic analysis

We performed biogeographic analysis to describe the distribution and colonization scenarios within the genus *Nothobranchius*. We used the Statistical Dispersal Vicariance analysis (SDiVA; [61] for the ancestral area reconstruction and for detection of the possible dispersal events. The t-files from the Bayesian analyses were used as input trees for the statistical part of the analysis (support value for the reconstructed ancestral area for the particular nodes).

The distribution matrix was given based on the presence of the species in the biogeographic region (or origin of the sample in some cases). The biogeographic scenario was obtained by mapping of the biogeographic areas (in S-DiVA) on the fully resolved phylogenetic tree originating from the MrBayes analysis (and produced by command: sumt contype = allcompat).

The biogeographic areas for the purpose of the analysis were mainly defined using the ecoregions proposed by [32] with some modifications. First, the largest ecoregion, “Coastal East Africa”, was divided into four parts – the coast, inland, island and North as most of the *Nothobranchius* species are distributed herein and our goal was to describe the more detailed view of the distribution patterns. Second, some adjacent ecoregions were joined into one area because they were not in the center of our interest, or there were represented only by few samples in our data set. Such reshuffling of these areas did not change any interpretation of the results. The areas (ecoregions) considered within this study were as follows (letter corresponds to the code used in analysis; see also map in Figure 2): A) Coastal East Africa (corresponds to ecoregion 564) – coast B) Coastal East Africa (ecoregion 564) – inland C) Coastal East Africa (ecoregion 564) – island D) Coastal East Africa (ecoregion 564) – Northern Mozambique E) Kenya Coastal drainages (ecoregion 567) F) Lower Zambezi (ecoregion 561 and 576) G) Lake Malawi basin (ecoregion 559) H) Western + Central Africa (ecoregions 506, 515, 520 and 522) I) Zambezi/Kafue/lakes (ecoregions 544, 557 and 558) J) Malagarasi (ecoregion 543). The maximum number of regions allowed in the ancestral area was set to three. This corresponds to the biological relevance, as *Nothobranchius* are species with very small areas having restricted ability to disperse. Therefore, allowing wider distribution in analysis would not have support in the natural history of the taxon. Further, the species with the widest recent distribution is *N. melanospilus* occurring in only two regions (A and C) and such constraint has been previously justified [62]. In general, *Nothobranchius* species have very limited distribution ranges linked to the alluvium of a single fluvial system [23] and there is no species distributed widely across the most of the regions.

### Diversification rate analysis

We performed test for potential shifts in diversification rate during evolution of the genus Nothobranchius. We used likelihood-based approach in TreePar [34] package for R, which tests for significant shifts in diversification rate based on the given tree topology and node ages. The ultrametric tree from the BEAST analysis (one individual per species) was used as input data for the analysis.

### Availability of supporting data section

The data set supporting the results of this article is available in GeneBankAccession numbers KJ179270-KJ179615 (see also Additional file 5: Table S1).

### Ethics

This work did not involve living animals. The Fritz Lipmann Istitute has a general authorization for Nothobranchius research released by the local authority in the State of Thuringia (Veterinär- und Lebensmittelüberwachungsamt). The Scuola Normale Superiore has a general authorization for Nothobranchius research released from the Italian Ministry of Health (96/2003-A).

## Additional files

Additional file 1: Figure S1 Saturation plots for 1rst, 2nd and 3rd codon of COI. Additional file 2: Figure S2 Individual gene trees for COI, GLYT1, MYH6, SH3PX3, GPR85. Additional file 3: Figure S3 Calibrated phylogeny of Cyprinodonts. Additional file 4: Table S3 Output of TreePar. Additional file 5: Table S1 Sample list with accession numbers. Additional file 6: Table S2 Primer sequences.
