# Supplementary material for: The strange case of East African annual fishes: aridification correlates with diversification for a savannah aquatic group?
Source: BMC Evol Biol. 2014 Oct 14;14:210. doi: 10.1186/s12862-014-0210-3 (PMC4209228; doi:10.1186/s12862-014-0210-3)
Supplement: Additional file 1: Figure S1 — Saturation plots for 1rst, 2nd and 3rd codon of COI. [file 12862_2014_210_MOESM1_ESM.pdf]

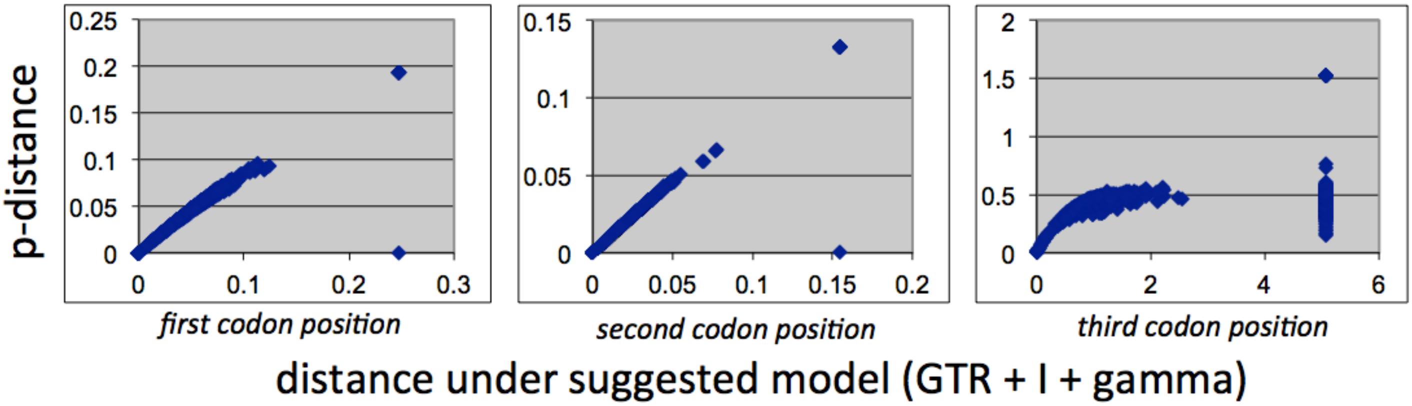

Figure S2: Distance plots showing evidence for saturation in the third position of codon in *COI* gene. The third position was therefore excluded from the analyses. The outlier points belong to outgroups.
