# Supplementary material for: The strange case of East African annual fishes: aridification correlates with diversification for a savannah aquatic group?
Source: BMC Evol Biol. 2014 Oct 14;14:210. doi: 10.1186/s12862-014-0210-3 (PMC4209228; doi:10.1186/s12862-014-0210-3)
Supplement: Additional file 2: Figure S2 — Individual gene trees for COI, GLYT1, MYH6, SH3PX3, GPR85. [file 12862_2014_210_MOESM2_ESM.pdf]

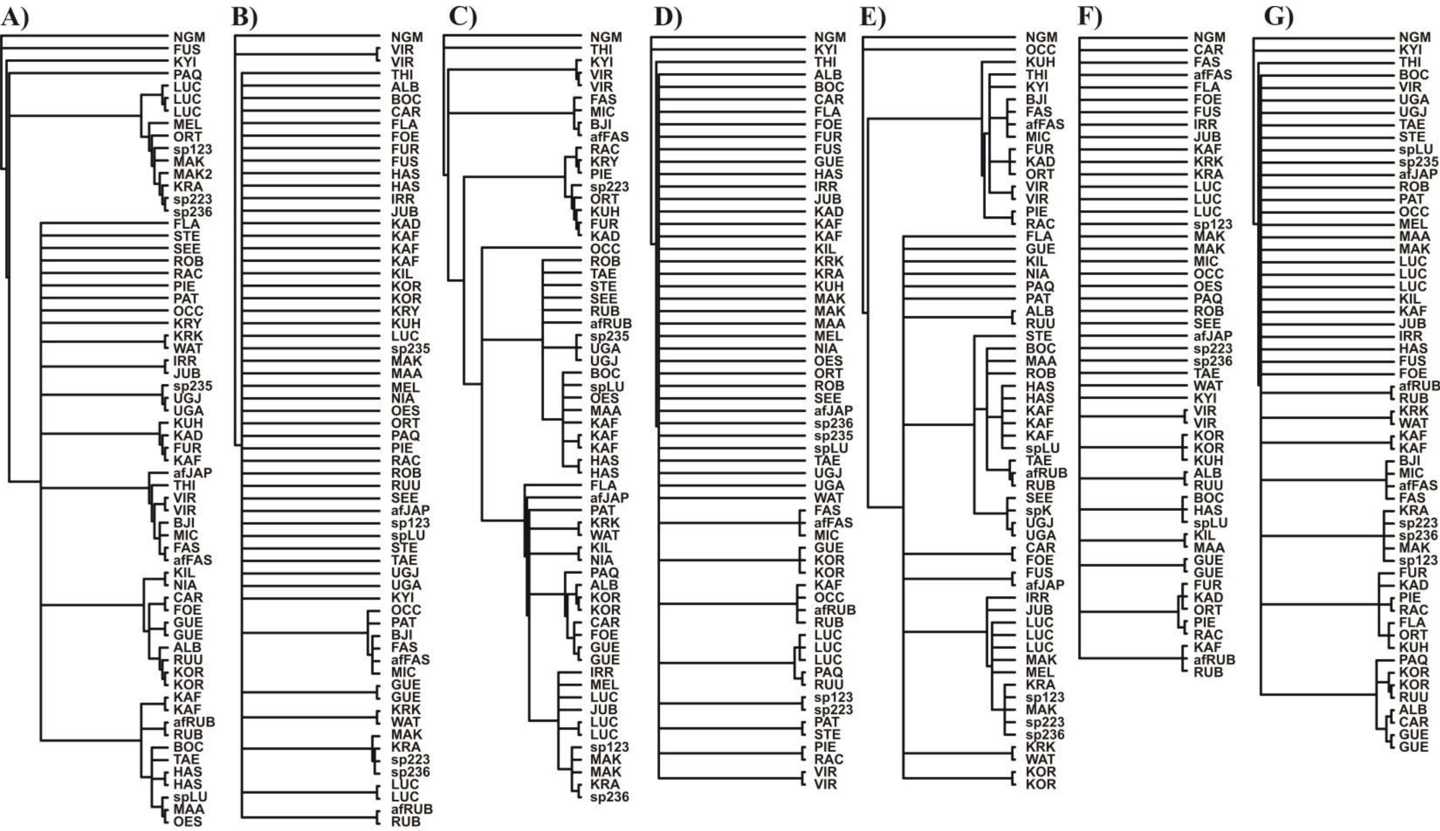


afFAS = *N.* aff. *fasciatus*
afJAP = *N.* sp. aff. *janpapi*
afRUB = *N.* aff. *rubroreticulatus*
ALB = *N. albimarginatus*
BJI = *N. bojiensis*
BOC = *N. boklundi*
CAR = *N. cardinalis*
FAS = *N. fasciatus*
FLA = *N. flammicomantis*
FOE = *N. foerschi*
FUR = *N. furzeri*
FUS = *N. fuscotaeniatus*
GUE = *N. guentheri*
HAS = *N. hassoni*
IRR = *N. interruptus*
JUB = *N. jubbi*
KAD = *N. kadleci*
KAF = *N. kafuensis*
KIL = *N. kilomberoensis*
KOR = *N. korthausae*
KRA = *N. krammeri*
KRK = *N. kirki*
KRY = *N. krysanovi*
KUH = *N. kuhntae*
KYI = *P. kyiawensis*
LUC = *N. lucius*
MAA = *N. malaissei*
MAK = *N. makondorum*
MEL = *N. melanospilus*
MIC = *N. microlepis*
NGM = *F. gardneri nigerianus*
NIA = *N. niassa*
OCC = *N. ocellatus*
OES = *N. oestergardi*
ORT = *N. orthonotus*
PAQ = *N. palmqvisti*
PAT = *N. patrizii*
PIE = *N. pienaari*
RAC = *N. rachovii*
ROB = *N. robustus*
RUB = *N. rubroreticulatus*
RUU = *N. ruudwildekampi*
SEE = *N. seegersi*
sp123 = *N.* sp. AG123
sp223 = *N.* sp. AG223
sp235 = *N.* sp. AG235
sp236 = *N.* sp. AG236
spLU = *N.* sp. "Lubumbashi"
STE = *N. steinforti*
TAE = *N. taenipygus*
THI = *F. thierryi*
UGA = *N. ugandensis*
UGJ = *N. ugandensis*
VIR = *N. virgatus*
WAT = *N. wattersi*

Figure S1: Results of phylogenetic analyses of individual genes. A) *COI* - all positions included, B) *COI* - only first and second position, C) *GLYT1*, D) *MYH6*, E) *SH3PX3*, F) *GPR85* and G) *ZIC1*.
