# Supplementary figures and images for: The strange case of East African annual fishes: aridification correlates with diversification for a savannah aquatic group?
Source: BMC Evol Biol. 2014 Oct 14;14:210. doi: 10.1186/s12862-014-0210-3 (PMC4209228; doi:10.1186/s12862-014-0210-3)

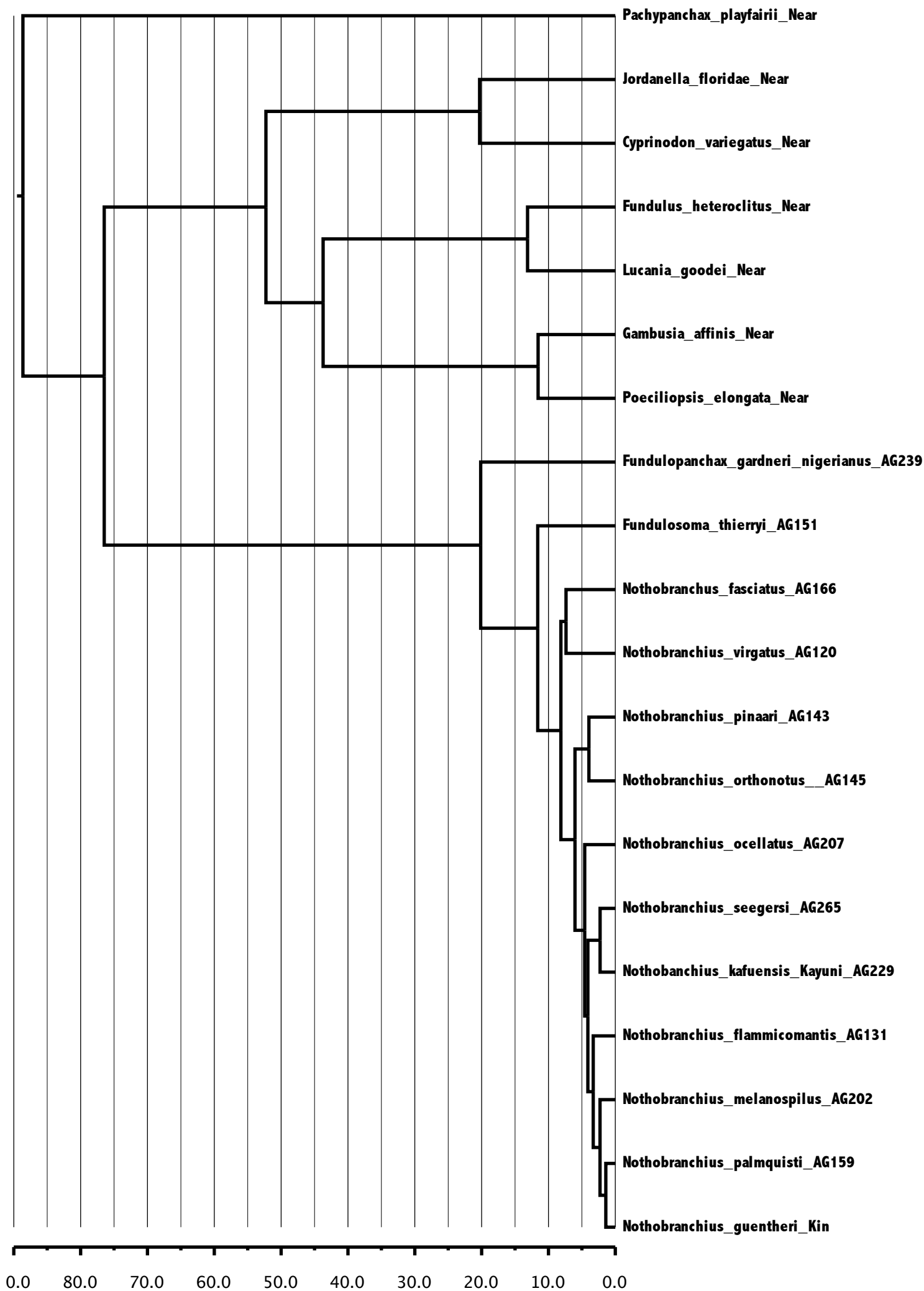

Supplement: Additional file 3: Figure S3 — Calibrated phylogeny of Cyprinodonts. [file 12862_2014_210_MOESM3_ESM.pdf]
