# Supplementary material for: The strange case of East African annual fishes: aridification correlates with diversification for a savannah aquatic group?
Source: BMC Evol Biol. 2014 Oct 14;14:210. doi: 10.1186/s12862-014-0210-3 (PMC4209228; doi:10.1186/s12862-014-0210-3)
Supplement: Additional file 6: Table S2 — Primer sequences. [file 12862_2014_210_MOESM6_ESM.docx]

| Primer-Number | Name | Gene | Sequence | T_m_^o^C |
| --- | --- | --- | --- | --- |
| 19 | cox1_for3 | COI | TTTCACA(AG)TATCAAACCCC | 51,3 |
| 20 | cox1_for4 | COI | ACAGGCTG(AG)ACAGTCTATCC | 58,3 |
| 21 | cox_rev3 | COI | AATGCTTCTCAGA(CT)AATAAA | 48,1 |
| 22 | Cox1_for5 | COI | AATCA(CT)AAAGATATCGGCAC | 52,2 |
| 23 | Cox1_rev4 | COI | AC(AT)GAAAG(AT)ACTTC(AGCT)CGTTT | 52,2 |
| 132 | nkcox1_1f | CoxI | GACCCAGCTGGWGGAGGAGA | 58 |
| 136 | nkcox1_1r | CoxI | CCTGCTAAGCCTAGGAAGTG | 54 |
| 55 | myh6_F459 | MyH6 | CAT(AC)TT(CT)TCCATCTCAGATAATGC | 57,6 |
| 56 | myh6_R1325 | MyH6 | ATTCTCACCACCATCCAGTTGAA | 58,9 |
| 57 | myh6_F507 | MyH6 | GGAGAATCA(AG)TC(GT)GTGCTCATCA | 60,6 |
| 58 | myh6_R1322 | MyH6 | CTCACCACCATCCAGTTGAACAT | 60,6 |
| 110 | Myh6_F1 | MyH6 | TCCAGTTCTTTGCCAGCATT | 55,3 |
| 111 | Myh6_R711 | MyH6 | CGACTTAGCCAGTGCACCA | 58,8 |
| 67 | Glyt_F559 | Glyt | GGACTGTC(AC)AAGATGACCAC(AC) | 60,3 |
| 68 | Glyt_R1562 | Glyt | CCCAAGAGGTTCTTGTT(AG)AAGAT | 58 |
| 69 | Glyt_F577 | Glyt | ACATGGTACCAGTATGGCTTTGT | 58,9 |
| 70 | Glyt_R1464 | Glyt | GTAAGGCATATA(GC)GTGTTCTCTCC | 61 |
| 108 | Glyt_F41 | Glyt | GGAAAGAATGAACATCACCTTG | 56,5 |
| 109 | Glyt_R777 | Glyt | CGGAGAGTTTAGCCTCACTAGA | 60,3 |
| 71 | SH3PX3_F461 | SH3PX3 | GTATGGT(GC)GGCAGGAAC(CT)TGAA | 61,2 |
| 72 | SH3PX3_R1303 | SH3PX3 | CAAACA(GT)CTC(CT)CCGATGTTCTC | 60,3 |
| 73 | SH3PX3_F532 | SH3PX3 | GACGTTCCCATGATGGC(AT)AAAAT | 58,9 |
| 74 | SH3PX3_R1299 | SH3PX3 | CATCTC(CT)CCGATGTTCTCGTA | 58,8 |
| 112 | SH3PX3_F5 | SH3PX3 | ACCCAACCAAGCAGACAAAG | 57,3 |
| 113 | SH3PX2_R555 | SH3PX3 | ATTTCCCAAACGCTGGAACT | 55,3 |
| 94 | Zic_I_F9 | Zic I | GGACGCAGGACCGCARTAYC | 63,5 |
| 95 | Zic_I_R967 | Zic I | CTGTGTGTGTCCTTTTGTGRATYTT | 59,7 |
| 96 | Zic_I_F16 | Zic I | GGACCGCAGTATCCCACYMT | 61,4 |
| 97 | Zic_I_R963 | Zic I | GTGTGTCCTTTTGTGAATTTTYAGRT | 58,5 |
| 140 | ZIC_I_F18 | Zic I | GCGCCTTCAAAATAAACCAC | 55,3 |
| 141 | ZIC_I_R693 | Zic I | AGTCCTCCCAGAAGCAGAT | 56,7 |
| 102 | Sreb2_F10 | Sreb2 | ATGGCGAACTAYAGCCATGC | 58,3 |
| 103 | Sreb2_R1094 | Sreb2 | CTGGATTTTCTGCAGTASAGGAG | 60,6 |
| 104 | Sreb2_F27 | Sreb2 | TGCAGGGGACCACAMCAT | 57,1 |
| 105 | Sreb2_R1082 | Sreb2 | CAGTASAGGAGCGTGGTGCT | 61,4 |

Table S2: The list of primers used in this study.
